# Delayed B-cell reconstitution in the bone marrow precedes the development of chronic graft-versus-host disease following pediatric hematopoietic stem cell transplantation

**DOI:** 10.3389/fimmu.2026.1923247

**Published:** 2026-09-16

**Authors:** Elisa Christine Nimskov-Peen, Klaus Müller, Katrine Kielsen, Hanne Vibeke Marquart

**Affiliations:** 1 Hematopoietic Stem Cell Transplantation and Primary Immune Deficiency, Department of Pediatric and Adolescent Medicine, Copenhagen University Hospital Rigshospitalet, Copenhagen, Denmark; 2 Department of Clinical Immunology, Copenhagen University Hospital Rigshospitalet, Copenhagen, Denmark; 3 Institute for Inflammation Research, Department of Rheumatology and Spine Disease, Copenhagen University Hospital Rigshospitalet, Copenhagen, Denmark; 4 Department of Clinical Medicine, Faculty of Health and Medical Sciences, University of Copenhagen, Copenhagen, Denmark; 5 Department of Immunology and Microbiology, University of Copenhagen, Copenhagen, Denmark

**Keywords:** alloimmunity and transplantation, B cells, B-cell reconstitution, biomarker, bone marrow, chronic graft-vs.-host disease, immune reconstitution, pediatric hematopoietic stem cell transplantation

## Abstract

**Introduction:**

Allogeneic hematopoietic stem cell transplantation (HSCT) in children remains challenged by chronic graft-versus-host-disease (cGvHD), significantly affecting morbidity and survival. While aberrant peripheral B-cell reconstitution has been implicated in cGvHD, B-cell development in the bone marrow (BM) remains less explored.

**Method:**

We investigated the B-cell reconstitution in BM samples and its potential to predict cGvHD in 76 children undergoing HSCT for acute leukemia (2010-2021) by blinded immunophenotypic analysis.

**Results:**

Children developing cGvHD exhibited delayed B-cell reconstitution in the BM with significantly lower proportions of total B cells and early B-cell precursors (BCP) I and II from +2 months to +9 months post-HSCT (all p<0.008) and reduced BCPIII and mature B cells at later time points compared to those without cGvHD (all p<0.05). In multivariable Cox proportional hazards models, low proportions of total B cells, BCPI, and BCPII at month +2 and month +3 were independent risk factors for cGvHD (HR=6.9-14.1, all p<0.017). At +2 months after HSCT, ROC analysis for predicting cGvHD with cut-off values of 0.16% (BCPI) and 0.99% (BCPII), yielded a sensitivity of 1.0 and a specificity of 0.69, while a cut-off value of 2.25% for total B cells resulted in a sensitivity of 0.90 and a specificity of 0.69.

**Discussion:**

These findings support previous evidence of disturbed B-cell reconstitution in cGvHD and suggest that phenotypic monitoring of BM B cells following pediatric HSCT may facilitate early cGvHD risk stratification. As BM assessments are routinely performed after HSCT, such monitoring could be readily integrated into existing clinical workflows.

## 1 Introduction

Allogeneic hematopoietic stem cell transplantation (HSCT) has markedly improved survival in children with high-risk acute leukemia (1), but long-term mortality and morbidity outcomes remain challenged by delayed or dysfunctional immune reconstitution (2). Chronic graft-versus-host disease (cGvHD) occurs in up to one-third of these patients (3, 4) and is a leading cause of non-relapse mortality, organ dysfunction and impaired quality of life both in children and in adolescents (5–7). cGvHD is a potentially severe heterogeneous complication, primarily affecting the skin, oral cavity, gastrointestinal tract, genitals, and/or lungs (8). Despite advances in immunosuppressive therapy, treatment responses remain suboptimal (9), and long-term immunosuppression increases the risk of relapse and severe infections. Importantly, diagnosis is still based on clinical criteria requiring overt organ involvement before recognition, and reliable biomarkers are lacking to predict cGvHD or to guide immunosuppressive treatment (10).

cGvHD is thought to evolve through three phases being initiated by early tissue injury and inflammation, followed by chronic inflammation with persistent immune dysregulation involving B- and T-cell immunity, ultimately resulting in aberrant tissue repair with fibrosis and irreversible organ damage (11). Growing evidence implicates disturbed B-cell homeostasis as a key player in the pathogenesis of cGvHD. Patients with cGvHD have increased circulating alloantibodies (12–15) and immunoglobulin G depositions in their skin (16) and lungs (17), correlating with cGvHD severity. Following HSCT, circulating B cells usually recover within 1–2 years with a reconstitution reflecting the normal B cell ontogeny. cGvHD development is preceded by a delay in the reconstitution of B cells in peripheral blood (PB) and a skewed distribution of B-cell subsets, including a reduction in regulatory B cells (3, 18–23).

In this study, we performed a detailed characterization of the B-cell reconstitution within the BM niche during the early post-engraftment phase up to 18 months after pediatric HSCT for acute leukemia and investigated the value of B-cell precursor profiles as a risk biomarker (24) for cGvHD.

## 2 Methods

### 2.1 Patient cohort

This study included children aged 1–18 undergoing their first allogeneic HSCT for acute leukemia between 2010 and 2021 at Copenhagen University Hospital (Rigshospitalet), Denmark. Eligible patients received BM or PB stem cell grafts from a human leukocyte antigen-A, –B, –C, -DRB1, and -DQB1 allele-matched donor. Of 89 eligible patients, 76 had available BM flow cytometric analyses of B-cell maturation markers and comprised the final cohort. The study was conducted in accordance with the principles of the Declaration of Helsinki and was approved by the Research Ethics and Research Support of the Capital Region of Denmark (#R-22057982).

### 2.2 Patient characteristics

The included children had a median age of 8.3 years (0.5-17.4) and were transplanted for acute lymphocytic leukemia (ALL) or acute myeloid leukemia (AML) (**Table 1**). All received myeloablative conditioning, with either total body irradiation (TBI; n=33) or chemotherapy alone (n=43). Stem cell sources were BM (n=72) or PB (n=4) grafts from matched sibling donors (MSD; n=17) or matched unrelated donors (MUD; n=59). GvHD prophylaxis consisted of cyclosporine A alone (n=14) or combined with methotrexate and anti-thymocyte-globulin (ATG; n=62). All patients engrafted. Patients were followed by flow cytometry for 18 months and clinically for >24 months post-HSCT; five patients were censored at the time of relapse or donor-lymphocyte infusion (day 161–454 after HSCT).

**Table 1:** Patient and transplant characteristics.

Characteristics	Pediatric cohort (n=76)	
*Diagnosis, no. (%)*	ALL	52 (68.4%)
	AML	24 (31.6%)
*Sex, no. (%)*	Female	23 (30.3%)
	Male	53 (69.7%)
*Recipient age (years), median (range)*		8.4 (0.5-17.4)
*Donor age (years), median (range)*		24 (3.1-56)
*Donor type (HLA-match)*	Matched sibling donor	16 (21.1%)
	Matched unrelated donor (10/10)	49 (64.5%)
	Matched unrelated donor (9/10)	11 (14.5%)
*Stem cell source, no. (%)*	Bone marrow	72 (94.7%)
	Peripheral blood stem cells	4 (5.3%)
*Myeloablative conditioning regimen, no. (%)*	*TBI-based (12 Gy)* TBI + VP16 TBI + Cyclophosphamide	35 (46.1%) 34 (97.1%) 1 (2.9%)
	*Chemotherapy based* Busulfan + Cyclophosphamide + Melphalan Busulfan + Thiotepa + Fludarabine Busulfan + Cyclophosphamide + VP16 Thiotepa + Fludarabine + Treosulfan Cyclophosphamide + VP16 + Treosulfan	41 (53.9%) 28 (68.3%) 9 (22.0%) 1 (2.4%) 2 (4.9%) 1 (2.4%)
*GvHD prophylaxis, no. (%)*	CyA alone	14 (18.4%)
	CyA + MTX	59 (77.6%)
	Tacrolimus + MTX	3 (3.9%)
*ATG, no. (%)*	No ATG	17 (22.4%)
	ATG	59 (77.6%)
*Female donor to male recipient, no. (%)*	No sex-mismatch	64 (84.2%)
	Sex-mismatch	12 (15.8%)

ALL, Acute lymphatic leukemia; AML, Acute myeloid leukemia; HLA, Human Leukocyte Antigen; Gy, Gray; TBI, Total body irradiation; ATG, Anti-thymocyte globulin; CyA, Cyclosporin A; MTX, Methotrexate.

### 2.3 Immunophenotyping of BM samples

Fresh BM samples were routinely examined for minimal residual disease (MRD) at +1, +2, +3, +6, +9, +12, +15, and +18 months post-HSCT (median 5 samples/patient, range: 1-8). Flow cytometry (6-8-color panels) was performed according to the patient’s diagnosis and protocol (NOPHO ALL2000, NOPHO-AML 2004, NOPHO ALL2008, NOPHO-DBH AML 2012, and ALLTogether guidelines) to detect leukemia-associated immunophenotypes. First-aspirate BM was used to avoid hemodilution. For this study, data were extracted and re-evaluated for B-cell subsets using two different panels (**Supplementary Figure 1**). We identified the following populations: Total B cells (CD19+), plasmablasts (CD19+CD38brightCD10-), B-Cell Precursor (BCP) stage I (CD45dimCD19+CD34+CD10bright), BCP stage II (CD45+CD19+CD34-CD10+CD20variable), BCP stage III (CD45+CD19+CD34-CD10dim- CD20+), and mature B cells (CD45brightCD19+ CD10-CD20+). Flow cytometry data were analyzed using FACSDiva software v. 8.0.2 with patient outcomes blinded.

### 2.4 B-cell concentrations in peripheral blood

Total PB B-cell concentrations were measured at +1 (n=59), +2 (n=61), +3 (n=66), +6 (n=67), +9 (n=55), +12 (n=58), +15 (n=27), and +18 (n=44) months post-HSCT. Absolute B-cell counts were determined in EDTA-anticoagulated blood using Trucount tubes (Becton Dickinson (BD), Denmark) with CD45-PerCP and CD19-PE monoclonal antibodies, analyzed by single-platform no-lyse-no-wash flow cytometry on a FC500 flow cytometer (Beckman Coulter, Denmark).

### 2.5 Acute and chronic GvHD

Acute GvHD (aGvHD) was prospectively diagnosed and graded by the treating physician according to the modified Glucksberg criteria. cGvHD was assessed by the treating physician at +3, +6, +9, +12, +18, and +24 months post-HSCT and when clinically indicated, according to the 2005 (25) or 2014 National Institutes of Health (8) consensus criteria adapted for pediatric purposes.

### 2.6 Statistical analysis

All B-cell proportions are reported as % of total mononuclear cells (MNC) in the BM. Changes over time in B-cell subsets were analyzed using the Wilcoxon signed-rank test, and associations with categorical variables using the Wilcoxon rank sum test. Spearman correlation assessed correlations with continuous variables. Logistic regression models evaluated associations between B-cell subsets and aGvHD, while Cox proportional hazard models assessed the predictive value of B-cell subsets and transplant characteristics for cGvHD. The discriminative ability of the Cox proportional hazard models was evaluated using Harrell’s concordance index (C-index) with 95% confidence intervals. Patients were censored at death, relapse, or re-transplantation if occurring before cGvHD onset. Kaplan-Meier curves and log-rank tests compared cGvHD incidence between groups. Sensitivity and specificity were determined from receiver operating characteristic (ROC) curves with cut-offs defined by the minimum distance to the upper-left corner of the unit square (sensitivity=1, specificity=1). Two patients without cGvHD were excluded from ROC analyses due to treatment-related death or relapse before 6 months post-HSCT. A two-sided p-value <0.05 was considered significant. Analyses were performed in R v4.3.0 (R Foundation for Statistical Computing, Vienna, Austria) and RStudio version v2024.04.2 (Boston, USA).

## 3 Results

### 3.1 B-cell reconstitution in BM following pediatric HSCT

Total B-cell proportions within the MNCs increased gradually from median 7.5% (range: 0.0-67.4) at month +1 to a maximum of 36.0% (range: 0.002-70.0) at month +9 (p<0.001) and remained stable until month +18 post-HSCT. Overall, the early B-cell compartment mainly comprised naïve BCPI and BCPII, however gradually declined as BCPIII and mature B cells increased during the following months (**Figure 1**).

**Figure 1:**
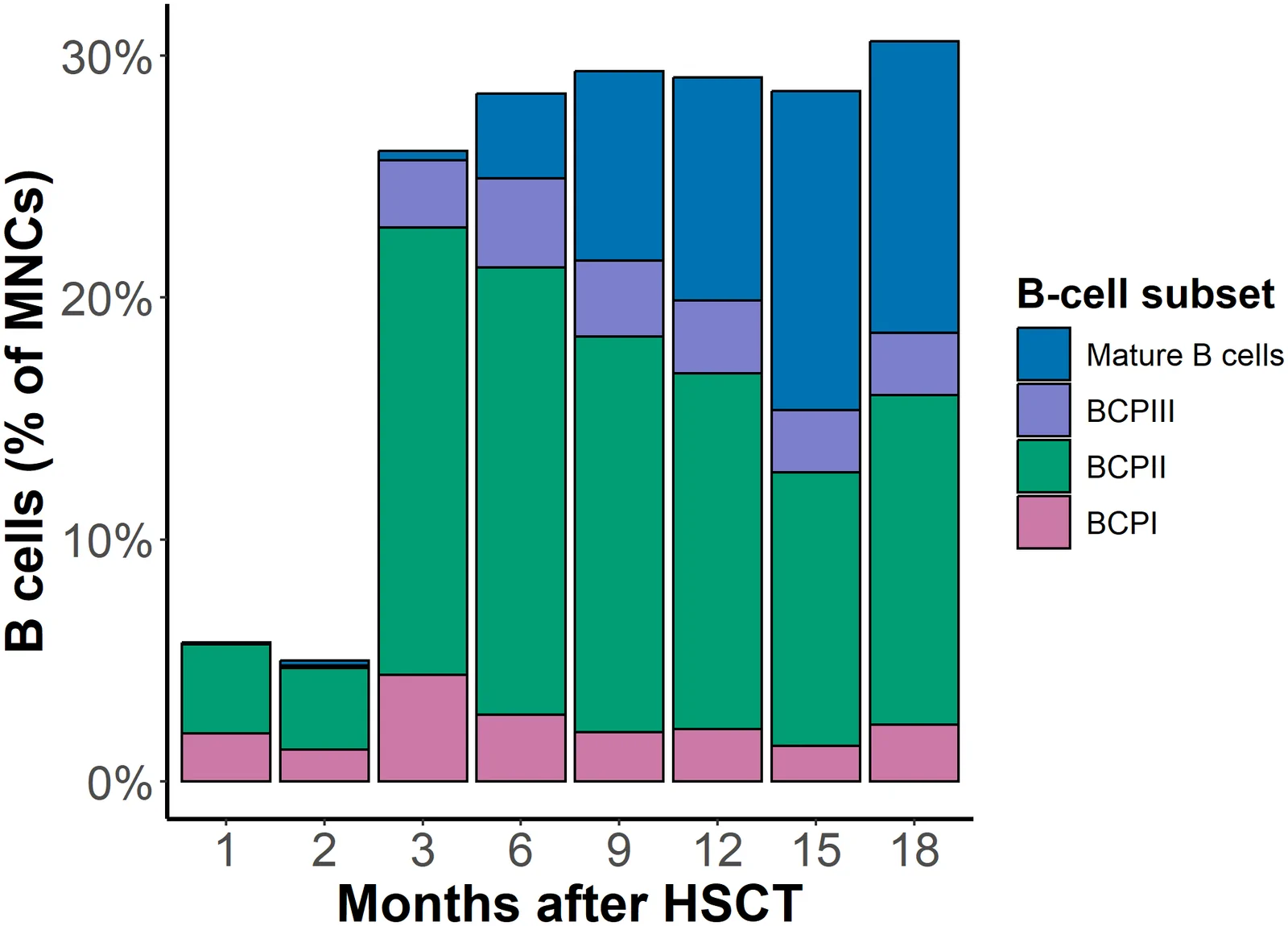
B-cell development and differentiation in the bone marrow of children following allogeneic HSCT. B-cell proportions (within mononuclear cells) generally increased in the bone marrow following HSCT, with a clear dominance of BCPI and BCPII at the earliest time points. From 3 months after HSCT, BCPII, BCPIII and mature B cells expand over time as B-cell maturation occurs. Stacked barplot showing median distributions of B-cell subsets for each time point. HSCT, hematopoietic stem cell transplantation; MNCs, mononuclear cells; BCP, B-cell precursor.

BCPI cells reached their maximum of 4.4% (range: 0.0-9.2) at month +3 and then tended to decrease to 2.4% (range: 0.7-6.9) at month +18, although not significantly (all p-values >0.05). In contrast, BCPII levels rose from median of 3.7% (range: 0.0-43.2) at month +1 to 18.5% (range: 0.0-59.3) at month +6 (p=0.003) reaching a plateau. BCPIII levels increased steadily from median 0.05% (range: 0.0-2.3) at month +1, peaking at month +6 with median 3.7% (range: 0.0-12.0) (p<0.0001). Mature B-cell levels rose gradually from median 0.02% (range: 0.0-0.4) at month +1 to 9.2% (range 0.4-23.1) at month +12 (p<0.0001), where it stabilized. Plasmablast levels in BM remained low, with medians between 0.0002-0.0004% (range: 0.0-0.7) at all time points (data not shown).

### 3.2 Associations between B-cell subsets and transplantation-related characteristics

Increasing age was associated with a delayed B-cell reconstitution, as higher recipient age correlated with lower total B-cell proportions at month +2 (r= -0.32, p=0.020) and +3 (r= -0.26, p=0.046), with fewer BCPI and BCPII in BM at month +3 (r= -0.36, p=0.005 and r= -0.26, p=0.049, respectively) as well as fewer BCPIII cells and mature B cells 12 months after (r= -0.40, p=0.026 and r= -0.41, p=0.023, respectively). Similarly, higher donor age correlated with fewer BCPI and BCPII cells at month +2 (r= -0.30, p=0.034 and r= -0.33, p=0.017, respectively), fewer BCPIII at month +3 and +6 (r= -0.35, p=0.016 and r= -0.41, p=0.007, respectively) and fewer mature B cells at month +6 to +12 (r= -0.55, p=0.0002 and r= -0.55, p=0.0008 and r=-0.54, p=0.002, respectively). Higher donor age also correlated with increased plasmablast proportions in BM at month +3 and +9 (r= 0.32, p=0.025 and r= 0.37, p=0.034, respectively).

Patients transplanted for AML had higher plasmablast proportions from month +2 (0.008% vs. 0.0002%) to +12 (0.002% vs. 0.0004%) compared with ALL (all p<0.030), except at month +9. TBI-based conditioning (used in 35/52 children with ALL) was associated with lower plasmablast proportions in BM from month +2 (0.0001% vs. 0.002%) to +6 (0.0001% vs. 0.001%) (all p<0.038), with a similar trend persisting at month +12 (0.0003% vs. 0.0006%, p=0.054).

Transplant with a MUD graft was associated with lower proportions of BCPIII (3.4% vs. 5.9%, p=0.009) and mature B cells (3.2% vs. 7.8%, p=0.041) at month +6 post-HSCT compared with MSD. Female donor-male recipient mismatch was associated with lower plasmablast proportions at month +6 post-HSCT compared with no sex mismatch (0.00007% vs. 0.0004%, p=0.038).

Graft source (PB vs. BM), nucleated cell dose, and use of ATG in the conditioning regimen were not associated with differences in the reconstitution of total B cells or B-cell subsets in the BM.

### 3.3 Acute GvHD is associated with changes in BCP proportions in the BM

A total of 44 children (57.8%) developed aGvHD with a median onset at day +17 (range +7 to +74). Of these, 15 (19.7%) developed grade I, 24 (31.6%) developed grade II, and 5 (6.6%) developed grade III-IV aGvHD. AGvHD grade II-IV was not associated with recipient or donor age, diagnosis, TBI-based conditioning, ATG, donor type, female donor-male recipient mismatch, or graft type (all p≥0.05).

Patients with aGvHD grade II-IV had lower proportions of total B cells and most B-cell subsets in the BM early after HSCT compared with grade 0-I, including reduced total B cells at month +1 to +3, and fewer BCPI, BCPII, BCPIII and mature B cells at month +2 and +3 (all p<0.026 and p<0.048, respectively) (**Figures 2A, B**). The reduction in BCPs did not persist beyond 3 months (all p≥0.05), whereas mature B-cell proportions remained reduced until +9 months after HSCT (all p<0.027). Plasmablast proportions were not associated with aGvHD grade II-IV at any time point.

**Figure 2:**
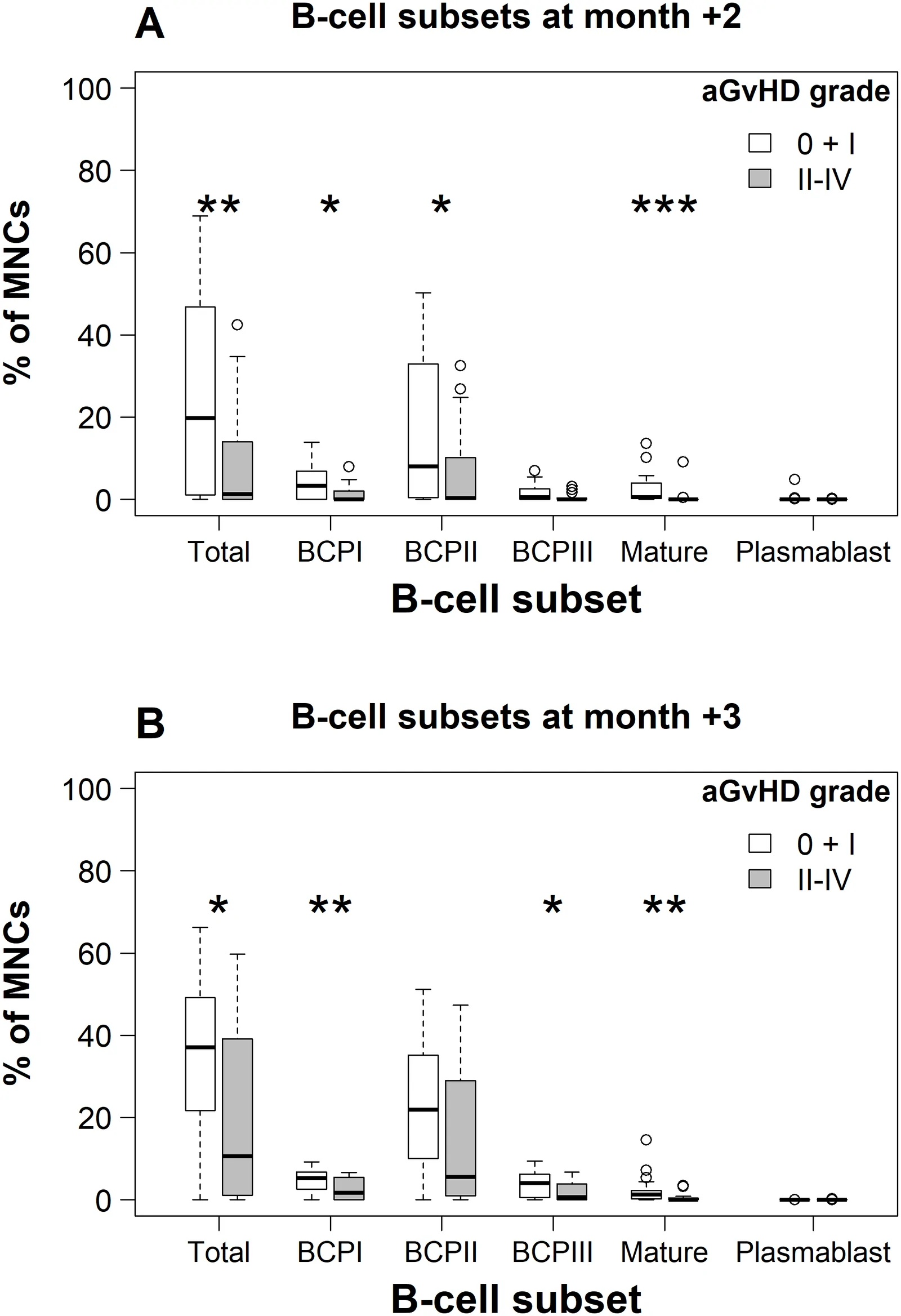
B-cell development in the BM stratified according to the development of aGvHD in 76 children following HSCT. Distribution of B-cell subsets in the BM at 2 **(A)** and 3 **(B)** months after HSCT in patients with aGvHD grade 0-I (n=47) and aGvHD grade II-IV (n=29). Boxplots show median and interquartile ranges. Statistical analyses show comparison of B cell levels between the two groups (*P<0.05, **P<0.01, ***P<0.001).

Multivariable logistic regression analyses confirmed that higher proportions of total B cells and early BCPs in BM were associated with lower odds of aGvHD grade II–IV at months +2 and +3 when adjusting for recipient and donor age. B-cell variables were log_2_-transformed, such that odds ratios represent the change in odds associated with a doubling of the B-cell proportion (**Supplementary Table 1**).

### 3.4 Delayed B-cell reconstitution in BM precedes cGvHD development

Thirteen children developed cGvHD with median onset of 7.1 months post-HSCT (range 4.0-12.9), of which four developed mild, four developed moderate and five developed severe disease.

B-cell reconstitution was significantly delayed in children developing cGvHD with fewer total B cells in the BM from +2 months to +9 months after HSCT compared with patients without cGvHD (**Figure 3A**, all p<0.008). Patients who later developed cGvHD also showed noticeably impaired early B-cell reconstitution, with nearly undetectable levels of the earliest BCP stages (BCPI and BCPII) at month +2 and significantly lower levels until +9 months post-HSCT compared with patients without cGvHD (all p<0.006, **Figures 3B, C**).

**Figure 3:**
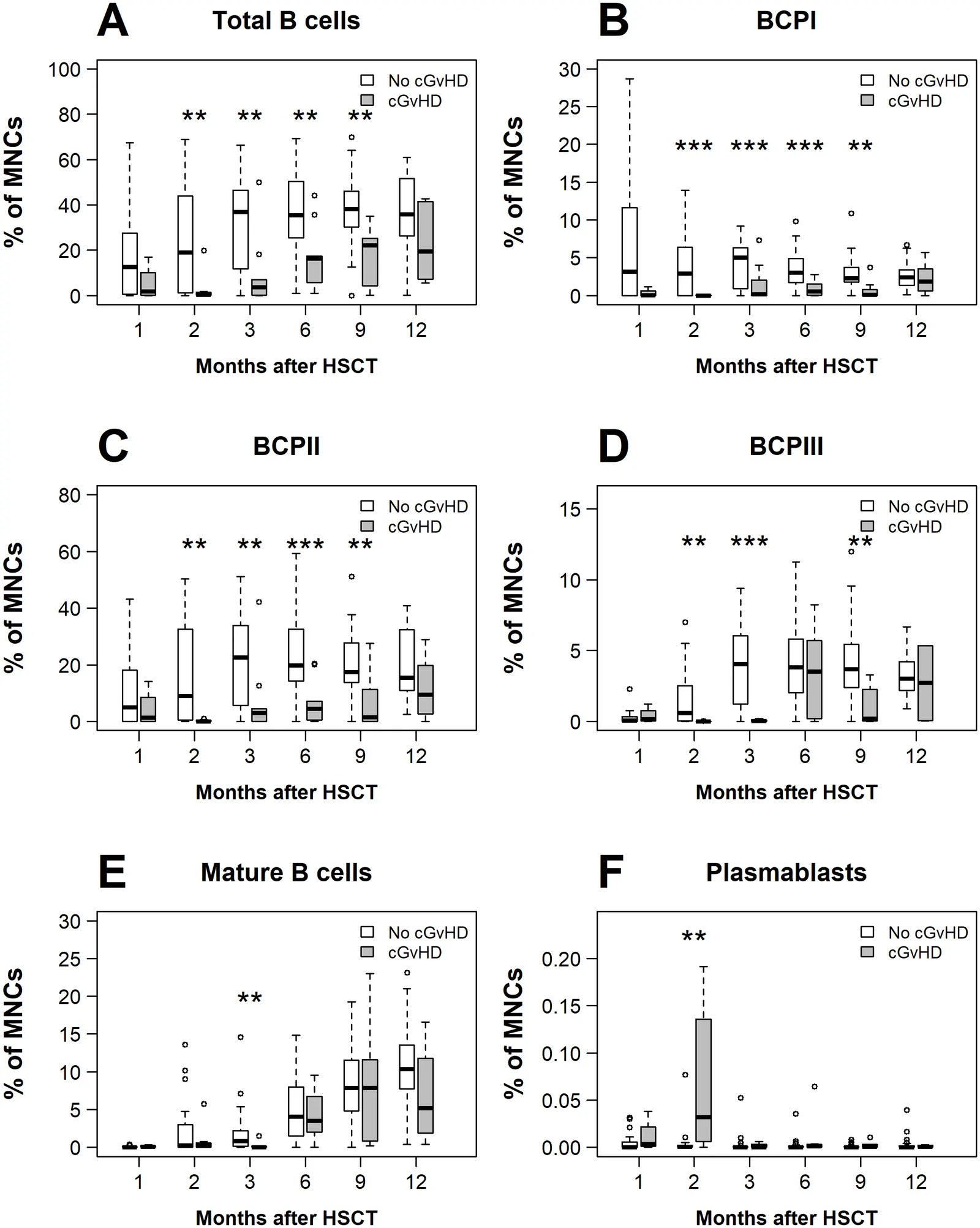
Delayed B-cell reconstitution in bone marrow precedes the development of cGvHD following pediatric allogeneic HSCT. **(A–F)** Distribution of B-cell subsets in bone marrow shown as proportion of total mononuclear cells (% MNCs). Statistical analyses show comparison of B-cell levels between patients who develop cGvHD (n=13) or do not develop cGvHD (n=63) (*P<0.05, **P<0.01, ***P<0.001, ****P<0.0001). Boxes show median and interquartile ranges for the total B-cell population **(A)**, B-cell precursor (BCP) stage I (CD45dimCD19+CD34+CD10bright) **(B)**, BCPII (CD45brightCD19+CD34-CD10+CD20+) **(C)**, BCPIII (CD45+CD19+CD34- CD20+) **(D)**, mature B cells (CD45brightCD19+CD10-CD20+) **(E)**, and plasmablasts (CD19+CD38+CD10-) **(F)**.

The pattern persisted as B-cell maturation advanced, as children who developed cGvHD showed delayed reconstitution of later precursor stages with lower proportions of BCPIII at months +2, +3, +9, +15 and +18 post-HSCT, and fewer mature B cells at months +3 and +15 post-HSCT (all p<0.044, **Figures 3D, E**). Plasmablast proportions in the BM were low overall, but higher at month +2 in patients who developed cGvHD compared with those who did not (0.03% vs. 0.0005%, p=0.004, **Figure 3F**).

As aGvHD is a known risk factor for cGvHD (25–27) and was associated with delayed early B-cell recovery in our cohort, we further performed a subgroup analysis restricted to patients with aGvHD grade II-IV (n=29). The aim was to explore whether early B-cell recovery differed between patients with aGvHD grade II-IV who later developed cGvHD (n=6) and those who did not (n=23). Here we observed trends of lower proportions of total B cells, BCPI, BCPII and BCPIII from month +2 to +12 as well as higher plasmablast proportions from month +1 to +9 in patients with aGvHD grade II-IV who later developed cGvHD compared with patients who did not (**Supplementary Figures 2A–D**). However, statistical power was limited by the small sample size and most differences did not reach statistical significance, except BCPII levels at month +6 (21.04% vs. 5.38%, p<0.045) and plasmablasts levels month +2 (0.044% vs. 0.000046%, p=0.012). These findings should therefore be regarded as exploratory, rather than a definite clinical conclusion and require further confirmation in a larger cohort.

### 3.5 B-cell subsets in BM may identify children at risk of cGvHD

Next, we analyzed whether B-cell subsets at month +2 and +3 or well-known transplant-related risk factors were predictive of cGvHD development (**Table 2**).

**Table 2:** Univariable Cox proportional hazards models assessing risk factors for developing chronic GvHD.

Variables		Risk of cGvHD		
		HR (95% CI)	*P-*value	Harrell’s C-index (95% CI)
*Characteristics*	Recipient age (per year)	1.0 (0.9 - 1.2)	0.58	0.55 (0.41-0.69)
	Donor age (per year)	1.0 (1.0 – 1.1)	0.72	0.54 (0.37-0.70)
	AML diagnosis	4.2 (1.4 – 12.9)	0.01	0.67 (0.53-0.80)
	TBI-based conditioning	0.5 (0.1 – 1.5)	0.21	0.59 (0.46-0.72)
	ATG as part of conditioning	1.7 (0.4 – 7.8)	0.47	0.55 (0.46-0.64)
	Matched unrelated donor	1.6 (0.4 – 7.2)	0.54	0.54 (0.45-0.63)
	PB graft	1.9 (0.2 – 14.6)	0.54	0.52 (0.44-0.60)
	Female donor to male recipient	0.4 (0.1 – 3.0)	0.37	0.55 (0.47-0.63)
	Nucleated cell dose	0.9 (0.7 – 1.2)	0.47	0.53 (0.36-0.70)
	Prior aGvHD (grade II-IV)	1.4 (0.5 – 4.2)	0.54	0.53 (0.40-0.67)
*B-cell subsets in BM at month +2*	Total B cells (<2% of MNC)	14.1 (1.8 – 111.6)	0.012	0.75 (0.63-0.88)
	BCPI (<0.1% of MNC)	6.9 (1.5 – 32.7)	0.014	0.72 (0.60-0.85)
	BCPII (<0.5% of MNC)	8.6 (1.8 – 40.7)	0.0064	0.75 (0.63-0.87)
	BCPIII (<0.05% of MNC)	3.5 (0.7 – 18.0)	0.14	0.65 (0.46-0.83)
	Mature B cells (<1% of MNC)	2.9 (0.4 – 23.2)	0.33	0.58 (0.43-0.72)
	Plasmablasts (>0.05% of MNC)	6.9 (1.6 – 29.4)	0.009	0.66 (0.50-0.83)
*B-cell subsets in BM at month +3*	Total B cells (<25% of MNC)	12.7 (1.6 – 101.4)	0.017	0.75 (0.63-0.87)
	BCPI (<2.5% of MNC)	6.8 (1.4 – 32.7)	0.017	0.72 (0.58-0.86)
	BCPII (<5% of MNC)	11.96 (2.5 – 57.7)	0.002	0.78 (0.64-0.92)
	BCPIII (<5% of MNC)	3.6 (0.4 – 29.2)	0.23	0.61 (0.48-0.74)
	Mature B cells (<1% of MNC)	5.6 (0.7 – 45.7)	0.11	0.66 (0.52-0.80)
	Plasmablasts (>0.0005% of MNC)	2.3 (0.6 – 9.1)	0.24	0.59 (0.42-0.77)

For each variable, the table reports the hazard ratio (HR), 95% confidence interval (CI), and p-value for a doubling in the level of B-cell subsets. Harrell’s concordance index (C-index; 95% CI) is reported as a measure of the model’s discriminative ability. AML, Acute myeloid leukemia; TBI, Total Body Irradiation; ATG, Anti-Thymocyte Globulin; PB, Peripheral blood; aGvHD, acute Graft-versus-Host-Disease; BM, Bone Marrow; MNC, Mononuclear cells; CD19, CD19+ population; BCPI, B-cell precursor stage I (CD45dimCD19+CD34+CD10bright); BCPII, B-cell precursor stage II (CD45brightCD19+CD34-CD10+CD20+); BCPIII, B-cell precursor stage III (CD45+CD19+CD34- CD20+); “Mature” B cells, (CD45brightCD19+ CD10-CD20+); Plasmablasts, (CD19+CD38+CD10-).

In Cox proportional hazards models, patients with AML had a higher risk of cGvHD compared with ALL patients post-HSCT (HR = 4.2, 95% CI = 1.4-12.9, p=0.012, **Table 2**). No other transplant-related factors, including aGvHD grade II-IV or TBI, were associated with cGvHD (**Table 2**, all *p≥*0.05) in univariable analyses. As TBI was restricted to ALL patients, a multivariable analysis adjusting for TBI was performed, in which AML remained associated with a trend toward increased risk of cGvHD (HR = 6.9, 95% CI = 0.9-55.3, P = 0.069).

Furthermore, patients with low proportions of total B cells, BCPI, and BCPII in the BM at month +2 (total B cells<2%, BCPI<0.1%, and BCPII<0.5%) and +3 (total B cells<25%, BCPI<2.5%, and BCPII<5%) post-HSCT had a higher risk of cGvHD than those with higher B-cell proportions (all p<0.02, **Figures 4A–F**; **Table 2**). BCPIII and mature B cells at months +2 and +3 were not associated with cGvHD (data not shown). Adjusting for AML diagnosis in multivariable Cox proportional hazard models, total B-cells, BCPI and BCPII at month +2 post-HSCT independently increased the risk of cGvHD (total B cells: HR = 12.0, p=0.019; BCPI: HR = 6.2, p=0.021; BCPII: HR = 7.1, p=0.016), while AML did not (p=0.18, **Table 3**). At month +3, total B cells, BCPI and BCPII in the BM as well as AML diagnosis appeared associated with increased cGvHD risk (**Table 3**).

**Figure 4:**
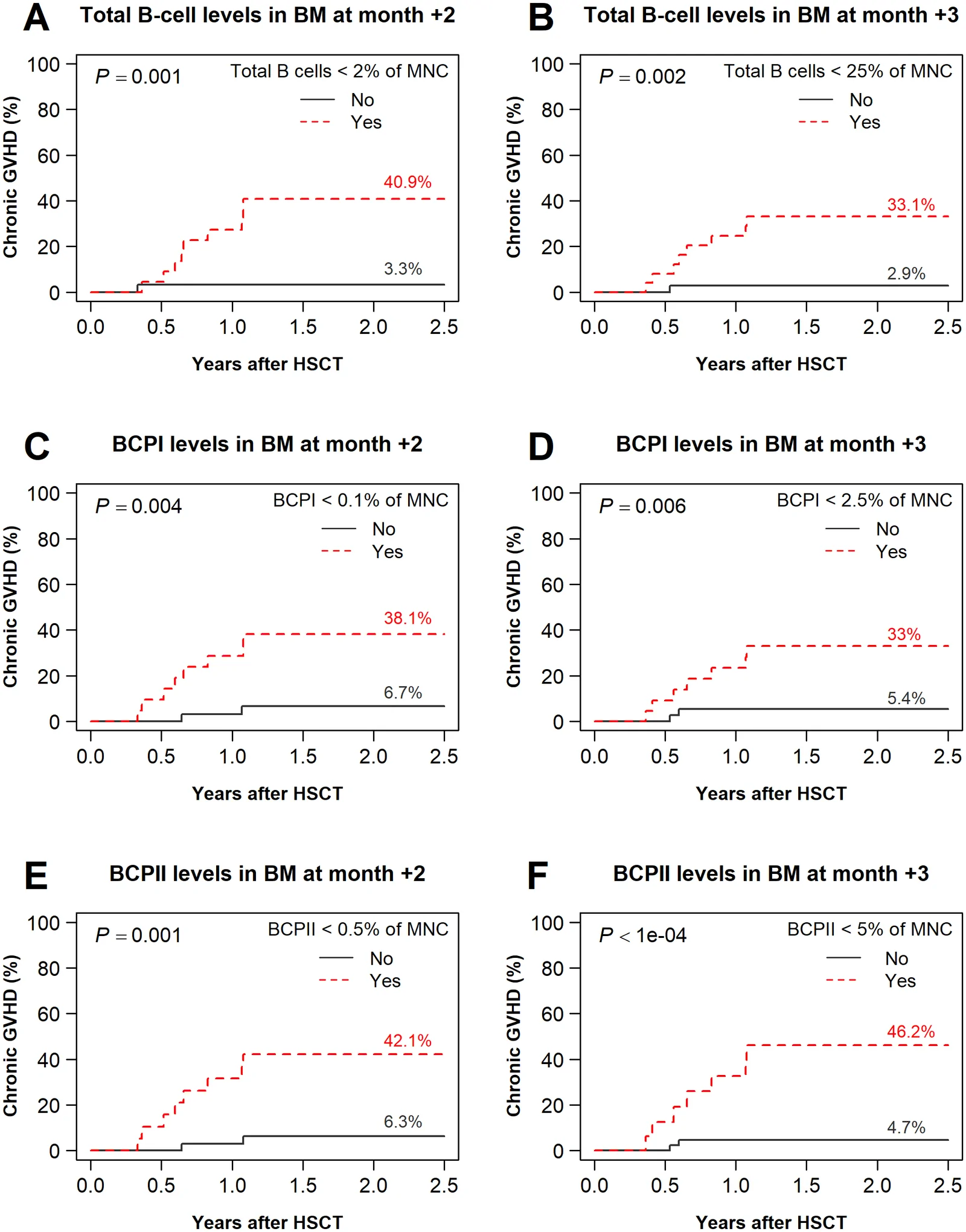
Early characterization of the B-cell department in the BM is predictive for development of cGvHD following HSCT. **(A–F)** Kaplan-Meier estimates showing the cumulative incidence of chronic graft-versus-host disease (cGvHD) among patients who had low proportions of Total B cells **(A, B)**, BCPI **(C, D)** and BCPII **(E, F)** within the mononuclear cells in the bone marrow at month +2 and +3 after allogeneic HSCT. The P-values indicate the difference between the two groups in two-sided log-rank tests.

**Table 3:** Multivariable Cox proportional hazards models analyzing B-cell subsets in the BM for developing chronic GVHD when adjusting for diagnosis.

Variables		Risk of cGvHD (*adjusted for diagnosis*)		
		HR (95% CI)	*P-*value	Harrell’s C-index (95% CI)
*B-cell subsets in BM at month +2*	Total B cells (<2% of MNC)	12.0 (1.5 – 96.3)	0.019	0.79 (0.65-0.94)
	BCPI (<0.1% of MNC)	6.2 (1.3 – 29.3)	0.021	0.79 (0.69-0.89)
	BCPII (<0.5% of MNC)	7.1 (1.4 – 34.6)	0.016	0.78 (0.68-0.89)
*B-cell subsets in BM at month +3*	Total B cells (<25% of MNC)	10.5 (1.3 – 84.6)	0.027	0.84 (0.74-0.93)
	BCPI (<2.5% of MNC)	4.7 (0.9 – 23.5)	0.059	0.83 (0.73-0.92)
	BCPII (<5% of MNC)	7.9 (1.5 – 40.5)	0.013	0.86 (0.79-0.93)

For each variable, the table reports the *adjusted* hazard ratio (HR), 95% confidence interval (CI), and p-value for a doubling in the level of B-cell subsets. Harrell’s concordance index (C-index; 95% CI) is reported as a measure of the model’s discriminative ability. BM, Bone Marrow; MNC, Mononuclear cells; Total B cells, CD19+ population; BCPI, B-cell precursor stage I (CD45dimCD19+CD34+CD10bright); BCPII, B-cell precursor stage II (CD45brightCD19+CD34-CD10+CD20+).

Next, we assessed whether applying the same cut-offs for total B cells, BCPI, and BCPII at months +2 and +3 as composite markers in a multivariable model improved cGvHD prediction. However, the composite markers were not superior to the individual markers, although they remained significantly associated with cGvHD risk (month +2: HR = 3.9, 95% CI = 1.1-13.9, p=0.035; month +3: HR = 11.96, 95% CI = 2.5-57.7, p=0.002).

To further explore diagnostic accuracy for predicting cGvHD, ROC curves were used to estimate sensitivity and specificity at the optimal cut-off values for B-cell subsets at month +2 and +3 post-HSCT. At month +2 post-HSCT, cut-off values of 0.16% (BCPI) and 0.99% (BCPII) yielded a sensitivity of 1.0 and specificity of 0.69 for predicting cGvHD, while a cut-off of 2.25% for total B cells resulted in a sensitivity of 0.90 and specificity of 0.69 (**Supplementary Table 2**). At month +3, a cut-off value of 19.7% for total B cells showed a sensitivity of 0.89 and specificity of 0.76 for predicting cGvHD, whereas BCPI and BCPII were less accurate (**Supplementary Table 2**).

Based on our findings, we propose a clinical workflow illustrating how BM B-cell immunophenotyping at +2 months after pediatric HSCT could be incorporated into early cGvHD risk stratification using the cut-off values identified in our Cox proportional hazards models (**Figure 5**).

**Figure 5:**
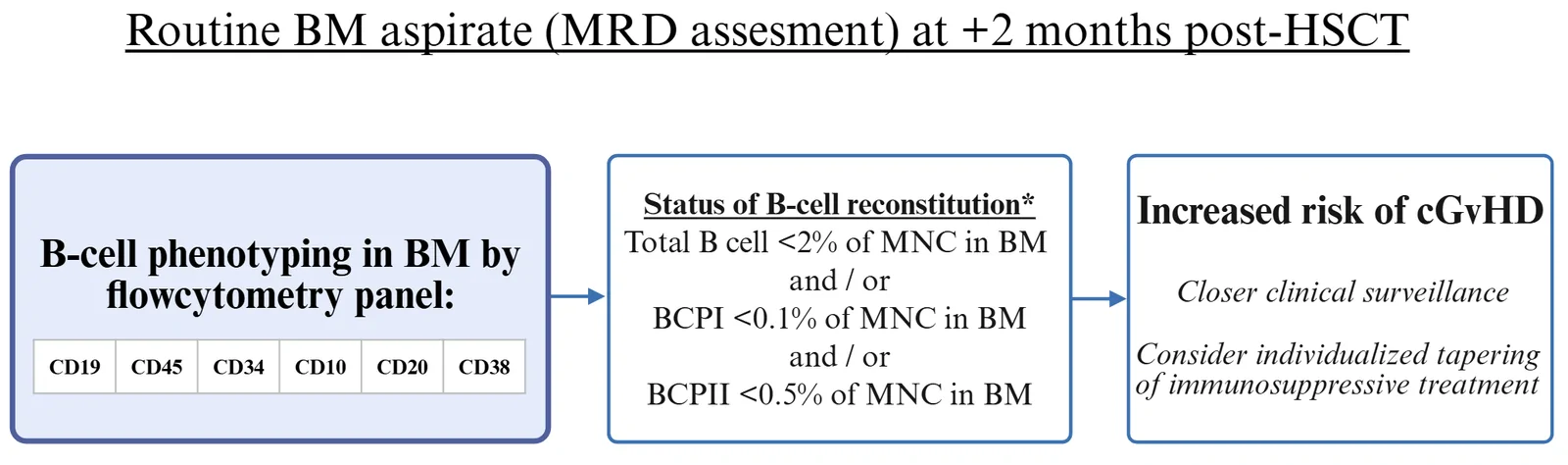
Proposed clinical workflow for early cGvHD risk stratification following pediatric HSCT. Patients with evidence of delayed BM B-cell reconstitution are identified as being at increased risk of developing cGvHD. These patients may benefit from closer clinical surveillance, and, pending prospective validation, could be considered for a more individualized management of immunosuppressive therapy, including a more cautious tapering in selected high-risk patients. This proposed workflow illustrates one potential clinical application of the present findings and requires validation in independent prospective cohorts before implementation in clinical practice. *Delayed BM B-cell reconstitution is defined by the cut-off values identified at month +2 post-HSCT in the present study (total B cells <2% of mononuclear cells (MNC), BCPI <0.1% of MNC, and BCPII <0.5% of MNC). Created in BioRender. Peen, E. (2026) https://BioRender.com/qwp9q2y.

### 3.6 Correlation between B cells in BM and PB

Low B-cell counts in PB have previously been reported as a risk factor for cGvHD. Therefore, we compared B-cell reconstitution in BM with PB. Overall, total B-cell proportions in the BM correlated positively with same-day PB B-cell concentrations from +2 to +18 months post-HSCT (all p ≤ 0.01) with the strongest correlation at the earliest time point (month +2: r=0.68, p<0.0001), **Supplementary Table 3**).

Age appeared to be the main factor influencing total B-cell numbers in PB as higher recipient age correlated with fewer total B cells from month +2 to +18 post-HSCT (all r< - 0.27, p<0.040), and higher donor age with fewer total B cells at month +3 to +18 post-HSCT (all r< -0.32, p<0.040). MUD transplantation was associated with lower total B-cell counts in PB at months +6 (0.21 vs. 0.30 x 10^9^ L, p=0.052) and +9 (0.29 vs. 0.64 x 10^9^ L, p=0.010) compared to MSD. Children with aGvHD grade II-IV had lower PB B-cell counts from months +1 to +6 after HSCT than those with aGvHD grade 0-I (all p<0.010, **Supplementary Figure 3A**). Diagnosis, TBI-based conditioning, and sex mismatch were not associated with differences in PB total B-cell levels.

Total PB B-cell concentration increased significantly from month +1 to +2 and onward in patients without cGvHD (all p<0.0001), whereas in those who developed cGvHD, a significant increase was not observed until month +6 (all p<0.031). PB B-cell counts were significantly lower in patients who developed cGvHD only at month +3 post-HSCT compared with those without cGvHD (0.004 vs. 0.045 x 10^9^ L, p=0.002, **Supplementary Figure 3B**). Cox regression model confirmed that lower PB B-cell counts at month +3 (<0.02 x 10^9^/L) increased the risk of cGvHD (HR = 6.8, 95% CI = 1.5-30.7, p=0.013). This association remained significant when adjusting for *either* AML diagnosis, which increased risk of developing cGvHD, recipient age, donor age, donor type, or prior aGvHD grade II-IV, which affected PB B-cell counts (data not shown).

## 4 Discussion

Biomarker-based strategies for predicting and preventing cGvHD are warranted to reduce long-term morbidity and mortality after HSCT, especially in children and adolescents with perspectives of long survival and with a risk profile involving impairment of normal growth potentially influenced by cGvHD and its treatment with corticosteroids (1). This study demonstrated that a delayed B-cell reconstitution in the bone marrow already 2 and 3 months after pediatric HSCT translated into an increased risk of developing cGvHD with both moderate-to-high sensitivity and specificity detectable almost 5 months before the median onset of cGvHD. To our knowledge, this is the first report to demonstrate the value of a detailed evaluation of the B-cell compartment in BM after HSCT to identify children at increased risk of developing cGvHD.

Our data demonstrate that B-cell reconstitution in children following HSCT largely recapitulates the physiological ontogeny of normal B-cell development in the BM, progressing from early precursor subsets toward more mature B cells during the first year after HSCT (28). In children who later develop cGvHD, this maturation trajectory appears globally delayed, with early B-cell subsets remaining significantly reduced throughout the first nine months compared with children who do not develop this complication. This pattern is consistent with observations from adult cohorts describing impaired early BCP recovery in the BM of patients who develop cGvHD (18–20, 29, 30). Notably, our data reveal a distinct alteration in B-cell maturation as early as two months post-HSCT—substantially earlier than most other reports. Using immunohistochemical staining of the B-cell lineage marker PAX5, Fedoriw et al. observed significantly reduced B-cell numbers in the BM at month +1 post-HSCT in adult patients who developed cGvHD compared with those who did not (30). Sánchez-García et al. reported significantly fewer Pre-B precursors (comparable to BCPII in our analysis) in BM from cGvHD patients obtained approximately seven months after HSCT in a mixed pediatric–adult cohort. Like our study, Sánchez-Garcia et al. found no differences in BCP levels +12 months post-HSCT between patients with and without cGvHD (18). Two additional studies found reduced total B cells and BCPs in the BM at +12 months after HSCT in adults with cGvHD compared to adults without cGvHD (20, 29), but did not examine earlier time points. However, firm conclusions from those studies are limited because time of cGvHD onset was not reported, making it difficult to conclude whether B-cell composition at this time point reflects altered immune reconstitution or the effects of cGvHD treatment. In general, biomarker studies in HSCT may be confounded by the effects of ongoing or prior medical and immunosuppressive treatment. As this is a retrospective observational study, our findings cannot establish causality. Nevertheless, the consistent association between impaired reconstitution across multiple B-cell subsets and the later development of cGvHD, with these abnormalities detected across several timepoints months before the onset of clinically apparent disease, supports the hypothesis that delayed B-cell reconstitution precedes, rather than merely reflects, the development of cGvHD.

Given that these alterations were detectable early post-HSCT and consistently preceded the onset of clinically apparent disease, we further evaluated the potential of B-cell subsets identified in the BM early post-transplant as risk biomarkers for cGvHD. In multivariable Cox proportional hazards models, low proportions of total B cells, BCPI and BCPII levels as early as 2 and 3 months after HSCT markedly increased the risk of developing cGvHD. In addition, ROC analyses showed nearly perfect sensitivity and a high specificity for BCPI and BCPII subsets at +2 months after HSCT, while total B cells at month +3 also held predictive potential with a high sensitivity and specificity combined. In our cohort, differences in total B cells and BCPs in the BM were associated with cGvHD at an earlier time point than changes in peripheral total B-cell counts. Furthermore, these differences were more consistent over time in the BM, suggesting that BM B-cell subsets may serve as more reliable risk biomarkers of cGvHD across multiple time points. While the observed predictive performance of our models was encouraging, the relatively small number of cGvHD events limits the robustness of these analyses and may lead to overfitting. The wide confidence intervals around several hazard ratio estimates indicate limited precision. Although Harrell’s C-indexes suggested moderate-to-good discrimination, validation in larger, independent cohorts will be necessary to confirm the reproducibility and generalizability of these findings. As regular MRD evaluations of BM are part of routine monitoring after HSCT for acute leukemia, early characterization of the B-cell compartment in the BM has the potential to be an easily accessible tool to aid in identifying children at risk of developing cGvHD and potentially guide tapering of immunosuppressive therapy, particularly by looking at the proportion of total B cells in the BM. A proposed clinical workflow illustrating the potential integration of BM B-cell immunophenotyping into routine post-transplant monitoring is shown in **Figure 5**. However, prospective validation in larger, independent cohorts will be essential before these biomarkers can be incorporated into clinical decision-making or used to guide individualized immunosuppressive management.

The requirement of donor B cells for the development of cGvHD has been demonstrated in murine models and was recently established in humans (3, 11, 31). In the current understanding of cGvHD pathogenesis, B cells contribute to chronic immune dysregulation by antigen presentation, antibody production, and promotion of tissue fibrosis through interactions with macrophages and fibroblasts (13). As suggested by our study and others, the gradual re-establishment of B-cell homeostasis after HSCT is profoundly disturbed in patients who develop cGvHD (13, 21, 25, 32, 33). It has been proposed that cGvHD is initiated by differentiation of BCPs in the BM into a pool of transitional B cells with autoreactive B-cell receptors (BCR) (3, 34), suggesting that even subtle disturbances in B-cell homeostasis, like a delay in B-cell reconstitution or impaired tolerance checkpoints (35, 36), could increase the risk of breaking B-cell tolerance (34). Furthermore, peripheral reduction of naïve B cells in patients developing cGvHD has been shown to cause sustained elevations of circulating B-cell activating factor (BAFF), likely reflecting reduced BAFF consumption (23, 25, 26, 37–42). Elevated BAFF levels promote survival of peripheral B cells, including autoreactive B cells (43–45) and augment BCR responsiveness and alloantibody production (46, 47), altogether fostering loss of peripheral tolerance. In line with previous findings (33, 37, 38), we found a remarkably lower concentration of circulating total B cells in patients developing cGvHD compared to those without cGvHD, although this difference was only significant at month +3 after HSCT. Importantly, peripheral CD19+ B-cell counts correlated strongly with reduced total B-cell proportions in the BM, suggesting that the low number of circulating B cells reflects a reduced B-cell production in the BM rather than peripheral destruction, consistent with earlier findings (20, 29). These results raise the question of whether resetting B-cell development in the BM could limit further disease progression in cGvHD, like how anti-CD19 CAR-T therapy efficiently resets aberrant autoimmunity through deep B-cell depletion in B-cell driven autoimmune diseases like systemic lupus erythematosus (48) and how rituximab improved symptom scores in patients with steroid-refractory cGvHD by depleting CD20^+^ B cells (49).

Although BAFF concentrations were not measured in our cohort, these findings support a model in which delayed BM B-cell reconstitution leads to reduced peripheral B-cell numbers, resulting in elevated systemic BAFF concentrations that may facilitate the survival and expansion of autoreactive B cells. Whether elevated BAFF also directly influences early B-cell maturation within the BM remains largely unexplored and cannot be addressed by our current data. Future studies combining longitudinal BM immunophenotyping with simultaneous BAFF measurements in both PB and BM will be important to clarify the contribution of BAFF to delayed B-cell reconstitution in the BM and subsequent cGvHD development.

The several strengths of this study include the extensive serial BM examinations conducted in a relatively large and homogeneous cohort of children transplanted for acute leukemia with an extensive characterization of the B-cell reconstitution after HSCT. However, it also has important limitations, particularly the low frequency of cGvHD and the observational design.

In conclusion, we demonstrate that a delayed B-cell reconstitution with a lack of BCPs in the BM as early as +2 and +3 months after HSCT is strongly associated with later development of cGvHD in a pediatric cohort. Our results further indicate an earlier and better predictive value of B-cell reconstitution when assessed in BM compared with PB. These data suggest that evaluation of B-cell subsets in the BM has the potential to be used as a risk biomarker for cGvHD, which could be easily implemented as part of routine MRD monitoring and potentially lead to individualized tapering of prophylactic immunosuppressive treatment and improved monitoring of patients at high risk of cGvHD, in the end hopefully minimizing the severity of cGvHD in children following HSCT.

## Data Availability

The raw data supporting the conclusions of this article will be made available by the authors, without undue reservation.
