# Supplementary material for: Delayed B-cell reconstitution in the bone marrow precedes the development of chronic graft-versus-host disease following pediatric hematopoietic stem cell transplantation
Source: Front Immunol. 2026 Sep 16;17:1923247. doi: 10.3389/fimmu.2026.1923247 (PMC13623726; doi:10.3389/fimmu.2026.1923247)
Supplement: Supplementary file 1 [file Table1.docx]

**Table S1. Multivariable logistic regression models analyzing the association between B-cell subsets in the BM of patients with aGvHD grade II-IV with patients with aGvHD grade I-IV, when adjusting for recipient and donor age**

Continuous B-cell subsets were analyzed after log₂ transformation. Odds ratios (OR) from represent the change in OR associated with a doubling of the biomarker level. OR are reported for both univariable (unadjusted) and multivariable (adjusted for recipient and donor age) logistic regression models. Total B cells: CD19+ population. BCPI: B-cell precursor stage I (CD45dimCD19+CD34+CD10bright). BCPII: B-cell precursor stage II (CD45brightCD19+CD34-CD10+CD20+).

| **Timepoint** | **Variable** | **Adjustment** | **OR  (95% CI)** | ***P* value** |
| --- | --- | --- | --- | --- |
| **Month +2** | Total B cells | Univariable | 0.71 (0.54–0.93) | **0.012** |
|  |  | *Multivariable* | *0.68 (0.50–0.92)* | ***0.013*** |
|  | BCPI | Univariable | 0.55 (0.35–0.89) | **0.015** |
|  |  | *Multivariable* | *0.55 (0.33–0.91)* | ***0.021*** |
|  | BCPII | Univariable | 0.74 (0.56–0.98) | **0.033** |
|  |  | *Multivariable* | *0.73 (0.54–0.99)* | ***0.043*** |
|  | BCPIII | Univariable | 0.46 (0.20–1.06) | 0.068 |
|  |  | *Multivariable* | *0.45 (0.19–1.06)* | *0.069* |
|  | Mature B cells | Univariable | 0.34 (0.12–0.95) | **0.039** |
|  |  | *Multivariable* | *0.31 (0.11–0.86)* | ***0.024*** |
| **Month +3** | Total B cells | Univariable | 0.71 (0.53–0.95) | **0.021** |
|  |  | *Multivariable* | *0.67 (0.48–0.94)* | ***0.019*** |
|  | BCPI | Univariable | 0.60 (0.38–0.95) | **0.030** |
|  |  | *Multivariable* | *0.51 (0.29–0.88)* | ***0.016*** |
|  | BCPII | Univariable | 0.71 (0.52–0.96) | **0.029** |
|  |  | *Multivariable* | *0.67 (0.47–0.96)* | ***0.028*** |
|  | BCPIII | Univariable | 0.59 (0.35–0.99) | **0.045** |
|  |  | *Multivariable* | *0.55 (0.30–1.00)* | *0.050* |
|  | Mature B cells | Univariable | 0.31 (0.12–0.81) | **0.017** |
|  |  | *Multivariable* | *0.28 (0.10–0.77)* | ***0.014*** |

**Table S2. Summary of ROC analyses of B-cell subsets for predicting chronic GvHD**

This table summarizes ROC analyses evaluating the ability of either total B cells (CD19), BCPI or BCPII measured at either +2 or +3 months after HSCT to predict later development of cGvHD. For each subset, the area under the curve (AUC) with optimal cut-off value, sensitivity and specificity are reported. Optimal thresholds were determined using the minimum distance to the upper-left corner of the ROC space. Two patients were excluded from the ROC analyses due to treatment-related death or relapse within 6 months post-HSCT.
BM: Bone Marrow. MNC: Mononuclear cells. CD19: CD19+ population. BCPI: B-cell precursor stage I (CD45dimCD19+CD34+CD10bright). BCPII: B-cell precursor stage II (CD45brightCD19+CD34-CD10+CD20+).

| **Parameter** | | **ROC AUC** | **Cut-off value** | **Sensitivity** | **Specificity** |
| --- | --- | --- | --- | --- | --- |
| B-cell precursor in BM at month +2 | Total B cells (% of MNC) | 0.78 | 2.25% | 0.90 | 0.69 |
|  | BCPI (% of MNC) | 0.83 | 0.16% | 1.00 | 0.69 |
|  | BCPII (% of MNC) | 0.82 | 0.99% | 1.00 | 0.69 |
| B-cell precursor in BM at month +3 | Total B cells (% of MNC) | 0.82 | 19.7% | 0.89 | 0.76 |
|  | BCPI (% of MNC) | 0.74 | 2.53% | 0.78 | 0.71 |
|  | BCPII (% of MNC) | 0.80 | 4.75% | 0.78 | 0.84 |

**Table S3. The proportion of total B cells in the BM correlates positively with same-day total B-cell concentration in peripheral blood at all time points from +2 to +18 months after HSCT**

Spearman’s rank correlation coefficients (r) were used to evaluate the relationship between peripheral B-cell counts (CD19+) and total B-cell proportions in the bone marrow (BM) of patients at months +1, +2, +3, +6, +9, +12, +15 and +18 following pediatric HSCT. Positive r values indicate that higher total B-cell proportions in the BM is associated with greater peripheral B-cell reconstitution. Two-sided p-values are reported for each time point.

| **Month(s) after HSCT** | **Spearman’s r** | ***P* value** |
| --- | --- | --- |
| 1 | 0.345 | 0.067 |
| 2 | 0.68 | **<0.0001** |
| 3 | 0.63 | **<0.0001** |
| 6 | 0.43 | **0.002** |
| 9 | 0.45 | **0.004** |
| 12 | 0.55 | **0.0009** |
| 15 | 0.66 | **0.010** |
| 18 | 0.75 | **0.001** |

**Figure S1 - Gating strategy for the two different panels used to characterize B-cell maturation in BM samples after pediatric HSCT**

*For the two tubes (A and B), we started by identifying mononuclear cells and singlets [1 and 2], before dividing the mononuclear cells into B-cell populations.*

**(A)** B-cell subsets were identified by the expression of CD45 and CD19 [3]. The CD19+ B lymphocytes were gated based on their expression of CD34 and CD38. Plasmablasts were defined as CD19+CD38+ [5]. Within the CD34+ B-cell population [4], B-cell precursor stage I was determined as CD45^dim^CD19+CD34+CD10^bright^ [6]. The CD34- and CD38- B-cell population was further divided into B-cell precursor stage II [8] and III [9] and [7] as well as mature B cells [10] based on their expression of CD10 and CD20. **(B)** The CD34+ hematopoietic progenitor population was identified [3] and further subdivided based on their CD33 and CD19 expression [3] to identify the CD34+CD33+ myeloid progenitors [4] and the CD34+CD19+ B-cell precursor stage I population [5]. Separately, the CD19+ population [6] was identified within the mononuclear cell fraction. Within the CD19+ compartment [6], B-cell precursor stage II cells (CD19+ CD45^dim^) were identified by subtracting B-cell precursor stage I [5] from [7]. Lastly, B-cell precursor stage III and mature B cells (CD19+ CD45^bright^) were identified as a combined population, by subtracting B-cell precursor stage I and II [7] from the CD19+ population [6].

A

B

**Figure S2 - B-cell reconstitution in bone marrow of patients with aGvHD grade II-IV precede following HSCT stratified by later development of cGvHD**

**(A-F)** Distribution of B-cell subsets in bone marrow shown as proportion of total mononuclear cells (% MNCs) in children with aGvHD grade II-IV stratified into patients who develop cGvHD (n=6) and patients who do not develop cGvHD (n=23). Boxes show median and interquartile ranges for the total B-cell population **(A)**, B-cell precursor (BCP) stage I (CD45dimCD19+CD34+CD10bright) **(B)**, BCPII (CD45brightCD19+CD34-CD10+CD20+) **(C)**, BCPIII (CD45+CD19+CD34- CD20+) **(D)**, mature B cells (CD45brightCD19+CD10-CD20+) **(E)**, and plasmablasts (CD19+CD38+CD10-) **(F)**. Statistical analyses compare the two groups with exact p-values are depicted in figure (all P-values <0.1 is shown).

**Figure S3. B cell concentration in peripheral blood and development of GvHD following pediatric HSCT**
(**A**) Concentration of total peripheral B cells in children with aGvHD grade 0-I (n=47) and aGvHD grade II-IV (n=29). Boxes show median and interquartile ranges. Statistical analyses show comparison between the two groups (*P<0.0, **P<0.01, ***P<0.001, ****P<0.0001). (**B**) Concentration of total peripheral B cells in children with cGvHD (n=13) and without cGvHD (n=63). Boxes show median and interquartile ranges. Statistical analyses show comparison between the two groups (*P<0.0, **P<0.01, ***P<0.001).
